# Treating Hyperuricemia: The Last Word Hasn’t Been Said Yet

**DOI:** 10.3390/jcm10040819

**Published:** 2021-02-17

**Authors:** Elisa Russo, Daniela Verzola, Giovanna Leoncini, Francesca Cappadona, Pasquale Esposito, Roberto Pontremoli, Francesca Viazzi

**Affiliations:** 1 Department of Internal Medicine, University of Genova, Viale Benedetto XV, 6, 16132 Genova, Italy; elisa24russo@gmail.com (E.R.); daverz@libero.it (D.V.); giovanna.leoncini@unige.it (G.L.); cappadona.francesca@gmail.com (F.C.); pasqualeesposito@hotmail.com (P.E.); roberto.pontremoli@unige.it (R.P.); 2 Internal Medicine Unit, IRCCS Ospedale Policlinico San Martino, Largo Rosanna Benzi 10, 16132 Genova, Italy; 3 Nephrologic Clinic, Sant’ Andrea Hospital, Via Vittorio Veneto 197, 19121 La Spezia, Italy; 4 Nephrology Unit, IRCCS Ospedale Policlinico San Martino, Largo Rosanna Benzi 10, 16132 Genova, Italy

**Keywords:** hyperuricemia, urate lowering treatment, chronic kidney disease

## Abstract

Gout as well as asymptomatic hyperuricemia have been associated with several traditional cardiovascular risk factors and chronic kidney disease. Both in vitro studies and animal models support a role for uric acid mediating both hemodynamic and tissue toxicity leading to glomerular and tubule-interstitial damage, respectively. Nevertheless, two recent well designed and carried out trials failed to show the benefit of allopurinol treatment on kidney outcomes, casting doubts on expectations of renal protection by the use of urate lowering treatment. With the aim of providing possible explanations for the lack of effect of urate lowering treatment on chronic kidney disease progression, we will critically review results from all available randomized controlled trials comparing a urate-lowering agent with placebo or no study medication for at least 12 months and report renal clinical outcomes.

## 1. Introduction

The relationship between hyperuricemia (HU) and chronic kidney damage is bidirectional. Although a reduction in glomerular filtration rate (GFR) can precede and lead to the development of hyperuricemia, increased serum uric acid (SUA) levels per se can adversely impact renal function [1,2,3]. Several pathogenic mechanisms have been investigated to support the causative role of uric acid. Experimental studies show increased SUA levels might mediate kidney damage promoting innate immune response [4], inflammation [5], oxidative stress [6], activation of the renin-angiotensin aldosterone system (RAAS) [7], endothelial dysfunction [8], proliferation of vascular smooth muscle cells (VSMC) [9], resulting in glomerulosclerosis and interstitial fibrosis [10].

While population-based association studies cannot prove causation, it is fair to report that several observational studies showed elevated SUA levels are strong and independent predictors of early GFR decline and albuminuria in a very large study population with and without diabetes [11,12].

**Figure 1:**
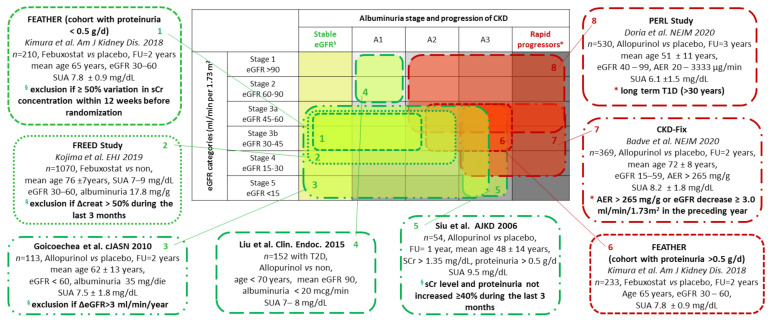
Schematic presentation of clinical characteristics of randomized controlled trial (RCT) patients among chronic kidney disease (CKD) categories. All available randomized controlled trials comparing a urate-lowering agent with placebo or no study medication for at least 12 months and reporting renal clinical outcomes (including kidney failure events or changes in glomerular filtration rate (GFR)) were included. The inclusion criteria (eGFR and Albuminuria categories using the NICE/KDIGO classification and the trend of kidney disease), the number of enrolled patients and baseline age, serum uric acid (SUA), eGFR and albuminuria levels were evidenced for each study. The meaning of renal disease stability or progression is specified for each study cohort. Trials with a positive or not evident renal effect attributable to urate lowering therapy (ULT) are colored with green and red, respectively. Abbreviations: CKD, chronic kidney disease; eGFR, estimated glomerular filtration rate (mL/min/1.73 m^2^); PERL, Preventive Early Renal Function Loss in Diabetes; CKD-Fix, controlled trial of slowing of Kidney Disease progression From the Inhibitionof Xanthine oxidase; AER, albumin excretion rate; FU, follow up; T1D, SCr, serum creatinine; type 1 diabetes; T2DM, type 2 diabetes; SUA, serum uric acid.

In intervention studies, xanthine oxidase inhibitors (XOIs) have been shown to reduce mean systolic and diastolic blood pressure in adolescents [13], and to improve endothelial dysfunction in specific subsets such as smokers [14] or patients with congestive heart failure [15]. While some small controlled clinical studies had previously suggested that urate lowering therapy (ULT) may retard chronic kidney disease (CKD) progression [16,17,18], more recent trials did not confirm a favorable effect of allopurinol on the evolution of kidney disease. In particular, in a randomized controlled trial (RCT) conducted in persons with type 1 diabetes (T1D) [19] and in the Controlled Trial of Slowing of Kidney Disease Progression from the Inhibition of Xanthine Oxidase (CKD-Fix) [20] carried out in patients with stage 3 or 4 CKD, SUA reduction by allopurinol was unable to modify the incidence of hard renal endpoints over a long time follow up. In order to reconcile these discordant results, and to identify the characteristics of patients most likely to benefit from renal protection, in this narrative review we will critically analyze the inclusion criteria and study design of all RCTs involving the use of ULT for at least 12 months and the availability of data on renal outcome (Figure 1).

## 2. Trials with a Positive Renal Effect Attributable to ULT

With the aim of selecting the best quality studies, we decided to include in this analysis only the RCTs that investigated the renal effect of a ULT compared to placebo or no treatment for at least 12 months. Therefore, despite the rising interest in the new anti-hyperuricemia drugs, studies analyzed in this review cannot include RCTs on uricosurics drugs and are forcibly limited to those on XOis (the old Allopurinol and the newer nonpurine selective XOi, Febuxostat). Topiroxostat, a new XOi approved for therapeutic use only in Japan, demonstrated a renoprotective effect by attenuating the reduction in eGFR in patients with diabetic nephropathy [21] and by inducing a 30% change of ACR in patients with renal impairment [22]. Unfortunately, RCTs on renal outcomes with Topiroxostat have a follow-up of no more than 28 weeks and therefore we did not include them in the present analysis.

We found that four studies were able to demonstrate a protective role of ULT [16,17,18,23,24] two studies were not [19,20] and one demonstrated a favorable effect on renal outcomes only in the subgroup of patients without proteinuria or with better initial kidney function [25]. As can be seen from Figure 1, the studies that demonstrated a protective effect of ULT were all characterized by similar inclusion criteria that guaranteed a substantial stability of renal function in the previous months [16,18,24,25] or a preserved eGFR above 60 mL/min at baseline [23]. Frequently they included patients with very low albumin excretion rate [23,24] and the presence of higher albuminuria levels judged a worse prognosis as success for the subgroup of FEATHER patients with proteinuria >0.5 g/day [25]. While some of these had a very small sample size [16,18,23] the FREED [24] is the largest RCTs available on this topic. These studies benefiting from ULT treatment include trials with both Allopurinol or Febuxostat vs. placebo or no study medication. Although Febuxostat can boast some preliminary data that suggest a greater renoprotective power than allopurinol, these findings derive from retrospective studies [26,27] or with a very short follow-up period [28].

## 3. PERL Study

The Preventing Early Renal Loss in Diabetes (PERL) trial enrolled 530 patients with T1D, SUA levels ≥ 4.5 mg/dL, mild to moderate increase in urine albumin excretion and eGFR 45–100 mL/min/1.73 m^2^ or significant GFR loss (>3 mL/min/1.73 m^2^/year) in the previous 3–5 years [19]. At baseline, the mean age was 51 years in the allopurinol and 52 years in the placebo group; the eGFR values were 75 and 74 mL/min/1.73 m^2^, respectively. Mean SUA decreased in the allopurinol group from 6.1 at baseline to 3.9 mg/dL during treatment whereas it remained at 6.1 mg/dL in the placebo group. Despite this sustained 36% reduction in SUA, the eGFR decreased at similar rates in the two treatment groups.

Several aspects that characterize the population recruited in this study could suggest that allopurinol therapy would hardly be able to confer renal protection to these patients. First of all, the PERL study includes patients with SUA ≥4.5 mg/dL, which is a very low cut-off to think that modulation of SUA may have a decisive impact on the progression of kidney disease in these patients. While results were similarly neutral in pre-specified subgroup analyses based on SUA levels (≤6.0 vs. >6.0 mg/dL), in the two trials by Siu and Goicoechea [16,17] showing favorable results in the arms randomized to allopurinol, the average SUA at baseline was significantly higher (9.5 and 6.7 mg/dL, respectively). 

Due to the well-known relationship between low eGFR and increased SUA levels, at least in part imputable to a decreased urate excretion rate, hyperuricemia and gout are frequently observed in patients with CKD [29,30]. Therefore, in a population of patients with CKD 2 and 3 with a median urinary albumin excretion rate in the range of microalbuminuria as those recruited in the PERL study, the level of SUA that would be expected to impact the progression of renal damage is higher than that observed in this study population.

Furthermore, patients included in the study showed a very long duration of diabetes. The 530 patients experienced a 34.6 years’ length of T1D, which obviously affected the natural history of their kidneys. The hyperfiltration distressing each individual nephron of these patients has inevitably triggered a vicious process leading to a progressive loss of nephronic mass that cannot be effectively countered with the ULT.

Furthermore, this population is largely represented by fast progressor patients as confirmed by the high slope of eGFR (about 2.5–3 mL/min/year) observed despite minimum or no renal damage at baseline. The FEATHER study provides a hint that febuxostat is presumably more effective for patients with less kidney damage such as those without proteinuria and those for whom serum creatinine concentration was lower than the median [25]. In a larger randomized study with a longer follow-up of about three years, Febuxostat showed a 25% reduction in the primary outcome mainly sustained by a reduced proportion of patients with a progression in albuminuria [24]. Results of the FREED study further support the view that renal protection may be more evident in the early stages of disease. In fact, in the latter study mean eGFR value was significantly better (55 mL/min, RAC 17 mg/g) than that recorded in the FEATHER study (eGFR 45 mL/min and RAC 120 mg/g). The potential of the protective effect of XOis seems to be supported by the very same data from PERL, wherein patients with normoalbuminuria seem to benefit more from treatment with allopurinol as compared to patients with a higher albumin excretion rate (although without reaching statistical significance).

In addition, the mean glycated hemoglobin level was 8.2 ± 1.3%, which means that a subset of patients was not at the target. As previously reported, severe hyperglycaemia may induce a reduction in SUA levels due to the uricosuric effect of sodium-glucose co-transporter-9 (SGLT9) activation secondary to increased glucose traffic in the tubular lumen. This mechanism leads to a J-shaped relationship between SUA and glycosylated hemoglobin, making it more complex to understand the relationship between the pharmacological modulation of urate and kidney damage.

Moreover, in the PERL study, blood pressure values were, on average, at target. This could account for the lack of nephroprotection by ULT observed in this trial. In fact, favorable cardiovascular (CV) and renal effects of SUA lowering treatment may be mediated by preventing endothelial dysfunction, vascular stiffness, and CV related events.

Finally, the large heterogeneity in the study population together with the small sample size makes it difficult to correctly interpret the data.

## 4. CKD Fix Study

The CKD-FIX randomized 369 patients to either allopurinol (*n* = 185) or placebo (*n* = 184) [20]. Eligible patients were adults with stage 3 or 4 CKD and albumin to creatinine ratio (ACR) ≥265 mg/g or eGFR decline rate ≥3.0 mL/min/1.73 m^2^ in the preceding 12 months. Overall, baseline age (62 years), eGFR (32 mL/min/1.73 m^2^), and SUA levels (8.2 mg/dL) were nearly identical in the two groups. The median urinary ACR (717 mg/g), the eGFR between 15 and 59 mL per minute per 1.73 m^2^, or its decrease of at least 3.0 mL per minute per 1.73 m^2^ in the preceding year, depict a figure of very high risk of progression. As discussed before, this is not the setting in which clinicians and researchers can expect the desired effect from ULT.

The trial, as clearly stated by the Authors in the limitation section, is underpowered as a result of incomplete enrollment and a high percentage of patients who discontinued the study regimen. In fact, in 20 months, recruitment reached 60% of the target number (i.e., 276 completed the trial, although the planned enrollment was 620 participants) and, based on trial logistics and funding, it was stopped by the steering committee. Moreover, during the study period, 54 patients (30%) in the allopurinol group and 45 patients (25%) in the placebo group discontinued the assigned regimen and a post hoc power calculation showed that the sample required to accommodate the discontinuation rate of 30% was 1006 patients. 

An interesting hypothesis is that the pathogenic role of SUA may be different in different CKD strata. In the CKD FIX, mean SUA levels remained constant in the placebo group and decreased in the allopurinol group to 5.1 mg/dL at 12 weeks and remained at 5.3 mg/dL along the study period with a ~35% reduction substantially superimposable to that observed in the PERL study. Nevertheless, the trial did not have a serum urate level-based inclusion criterion, and this contributed to the heterogeneity of the sample study with some participants with normal and others with elevated serum urate levels at enrollment.

Once again, while the CKD-FIX Study failed to demonstrate any benefit of allopurinol on renal functional decline, this lack of effect has several possible explanations, comprising the selection of an underpowered study population with a very high risk of progression. As a matter of fact, increased SUA levels have been proven to be more predictive of kidney disease progression in the early stages of CKD and in patients without proteinuria than in patients with more severe kidney damage [31] as is the case for patients recruited in the CKD-FIX trial.

## 5. Findings from Reduction of Endpoints in Non-Insulin-Dependent Diabetes Mellitus with the Angiotensin II Antagonist Losartan (RENAAL) and SGLT2i Trials Support the View That SUA May Be a Modifiable Risk Factor for Renal Disease

The gold standard for renal protection in CKD are RAAS-I and SGLT2-inhibitors although this has mainly been demonstrated in subgroups of patients with clinical proteinuria and/or diabetes. Interestingly Losartan (an Angiotensin II Receptor Blocker) and gliflozins share a potentially renal protective features in that they lead to increased urinary excretion of urates. Sodium-glucose cotransporter-2 (SGLT2) inhibitors block the reabsorption of glucose at the proximal convoluted tubule and the glycosuria that results causes uric acid to be secreted into the urine. A recent meta-analysis of more than 60,000 patients showed that adults randomly assigned to receive an SGLT2 inhibitor had significantly lower SUA levels as compared to those assigned to receive a placebo or comparator medication [32]. In a longitudinal study of 300,000 adults with type 2 diabetes mellitus, a relative risk reduction in gout of nearly 40% has been observed among patients newly prescribed an SGLT2 inhibitor compared with those newly prescribed a glucagon-like peptide 1 (GLP1) agonist [33]. While the exact mechanism, in addition to glycosuria, by which SGTL2 inhibitors are thought to reduce SUA levels is not well understood, the hypothesis that this effect is part of the CV and renal protection induced by SGLT2 inhibitors is evoked by the mediation analysis undertaken in EMPAREG OUTCOME, suggesting that changes in SUA mediated ~20% to 25% of the reduction in CV death and heart failure death seen with empagliflozin [34,35].

Similar data linking a reduction of SUA levels to a positive effect on CV and renal events derives from the RENAAL Trial. Treatment with the antihypertensive drug losartan lowers SUA probably by inhibiting URAT1, leading to reduced UA reabsorption at the tubular level. In a post hoc analysis of 1342 patients with type 2 diabetes mellitus and nephropathy participating in the RENAAL, approximately one-fifth of losartan’s renoprotective effect that has been proposed could be attributed to its effect on SUA [36].

## 6. Conclusions

While several clinical practice guidelines emphasize the usefulness of serum urate evaluation for risk stratification [37], the magnitude and importance of the SUA role in the pathogenesis of organ damage might vary and depend on the severity and duration of the underlying disease [38]. This hypothesis calls for the need of clarifying how hyperuricemia should be defined in the presence of CKD and when ULT might be prescribed for CV and renal protection in individuals with CKD.

In line with these concerns are the results of a very recent meta-analysis including 28 prospective, randomized, controlled trials assessing the effects of ULT for at least six months on CV or kidney outcomes [39]. Chen Qi et al. found that ULT was associated with the reduction of blood pressure and retardation of the decline in GFR overtime. The authors did not find benefits on clinical outcomes, including major adverse CV events, all-cause mortality, and kidney failure and once again results were conditioned by short follow-up or low quality of the trials. Also, the trials involved in this meta-analysis have significant heterogeneity related to the level of kidney function, underlying disease, and other conditions such as the usage of renin-angiotensin-aldosterone system inhibitors or significant dropout rate that could have confounded results.

In summary, although the two recently published RCTs were unable to provide the expected answers to our questions on the nephroprotective role of allopurinol, the analysis of the literature does seem to leave it open to the possibility of demonstrating the beneficial effect of ULT in future trials. Due to inclusion criteria or insufficient power, it was foreseeable that these RCTs showed no protective effect of allopurinol. Numerous data seem to suggest that the renal or vascular damage attributable to uric acid cannot regress once it has established itself. Accordingly, the presence of increased SUA levels at baseline not only predicted the development of hypertension, but also significantly blunted the decrease in blood pressure associated with lifestyle changes in children, suggesting that such children might have progressed to the irreversible phase and structural renal damage may have occurred [40]. At first, these changes are urate-dependent, but then they can trigger a self-reinforcing loop that is unresponsive to urate-lowering treatment. For this reason, patients with better preserved renal function and children might benefit more from an early ULT. Although the enrollment of patients at very low risk for progression of chronic kidney disease significantly limits the ability of a trial to show a treatment benefit, there is still much to be learned about the effect of hyperuricemia. Moreover, the health and economic burden of chronic kidney disease and its impact on increased cardiovascular risk [38] from early [41] to advanced stages of kidney function impairment [42], justify the effort to promote trials involving younger patients with earlier and less severe mild renal involvement, possibly still able to favorably respond to the reduction of uric acid levels. Therefore, while there are currently no robust data to support the routine use of pharmacotherapy for all patients with asymptomatic hyperuricemia, adequately powered, randomized, placebo-controlled trials with appropriate selection criteria are needed to determine whether specific patient groups could benefit from ULT.
